# Smallholder Farmer's Knowledge and Practice Towards Farm Biosecurity Measures to Prevent Infectious Diseases in Bahir Dar City, Ethiopia

**DOI:** 10.1002/vms3.70651

**Published:** 2025-10-15

**Authors:** Birhan Agmas Mitiku, Ayele Adugna Gedifew, Temsegen Sendekie Ayalew

**Affiliations:** ^1^ School of Veterinary Medicine Bahir Dar University Bahir Dar Ethiopia; ^2^ Faculty of Veterinary Medicine and Animal Science Federal University of Mato Grosso do Sul Campo Grande Brazil

**Keywords:** animal farm, biosecurity, knowledge, practice, smallholder

## Abstract

Proper biosecurity practice is crucial in animal farming to reduce the risk of diseases. The aim of this study was to assess smallholder farmers’ knowledge and practice towards farm biosecurity measures and associated factors among smallholder farmers. A total of 125 farms were randomly selected, and the biosecurity level of each farm was assessed using an observation checklist, questionnaire and a biosecurity score. Overall farm biosecurity knowledge of respondents was 50 (40%); they had good farm biosecurity knowledge recorded. The assessment of biosecurity practice levels of the study farms revealed that 62 (49.6%) of the farms had a good biosecurity score. Our multivariable analysis showed that gender, education level and main occupation status were revealed in the study as the risk factors that had a significant relationship with good farm biosecurity knowledge and practices. Male farmers recorded better knowledge (adjusted odds ratio [AOR] = 1.43, 95% confidence interval [CI] [1.12–2.56]) and practice (AOR = 1.34; 95% CI [1.06–1.36]) compared to female farmers. Educated farmers are more likely to have good knowledge (AOR = 2.05; 95% CI [1.56–1.99]) and practice (AOR = 2.05; 95% CI [1.31–2.10]) than illiterate ones. Regarding occupation, professionals had good farm biosecurity knowledge (AOR = 3.79; 95% CI [1.05–1.54]) and practice (AOR = 1.071; 95% CI [1.01–1.87]) than traders. In conclusion, the study revealed that biosecurity measures were not adequately implemented. Smallholder biosecurity measures, at village‐level operations, need to empower farmers to make better decisions. Integrating biosecurity into motivations and availability of short‐term awareness is crucial. Associating biosecurity and disease management with improved livestock productivity and economic benefits brings sustainable rewards.

## Graphical abstract

It was crucial to determine farmers’ current knowledge and practice of farm biosecurity in order to empower them to properly devise good strategies on how to improve adopting biosecurity practices.

The practice and knowledge among smallholder farmers were examined in farm biosecurity practices; thus, 40% of the respondents had documented good farm biosecurity knowledge, and 49.6% had good biosecurity practices. Occupation status, education level and gender were predictors of practices.

The study concluded that the biosecurity practice is not very much in place, and there is a necessity for integrating biosecurity in motivation and short‐term awareness.

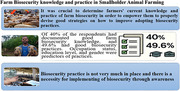

**Abbreviations**
AOR: adjusted odds ratio
CI: confidence interval
COR: crude odds ratio
FAO: Food and Agriculture Organization
OR: odds ratio

## 1 Introduction

Biosecurity is crucial for preventing the introduction and spread of diseases or pests on farms (Villarroel et al. 2007). It includes both external biosecurity, which targets preventing diseases from entering the farm, and internal biosecurity, which decreases the spread of disease within the herd (Moya et al. 2020). The implementation of biosecurity measures depends on the adoption of certain attitudes and behaviours. Technical knowledge is one of the key factors shaping farmers’ and veterinarians’ attitudes towards biosecurity, with studies highlighting how this knowledge affects decision‐making (Moya et al. 2020; Richens et al. 2018).

Maintaining optimal animal health is essential for productivity and safeguarding future livestock, as farms are often exposed to a number of pathogens from wildlife, pets or neighbouring animals (McElwain and Thumbi 2017). However, biosecurity implementation faces huge challenges, particularly in regions like Ethiopia, where poor farm management, inadequate health facilities, lack of prevention strategies and limited access to vaccines are prevalent (Tsegaye et al. 2023).

Inadequate knowledge and practices around biosecurity have contributed to the spread of diseases and the rise of antibiotic resistance (Bedekelabou et al. 2022; Waktole et al. 2023). For smallholder farmers, socio‐economic and literacy barriers further complicate the adoption of effective biosecurity measures (Gomez and Mbaga 2023). Effective biosecurity protocols, combined with vaccination, are critical for controlling disease transmission (Hyelda et al. 2020).

Diseases occur due to lack of proper care and management, inadequate nutritious feeding and some other factors, such as local disease burden. Generally, diseases can be defined as changes in the typical physical conditions of animals. Almost all types of animals can be affected by different types of diseases in their lifetime (Melkamu et al. 2016). Improper management of livestock production has recently become a source of concern, but also there is the rapid growth of pathogenic diseases and the impact on the development and spread of antibiotic resistance (Bedekelabou et al. 2022).

The effectiveness of the biosecurity practice depends on the control of illnesses at an early stage. According to Hyelda et al. (2020), illness transmission can be curbed through vaccination of the animals and biosecurity protocols. According to Abdurehman et al. (2023), biosecurity is a crucial procedure that guards against the deliberate and inadvertent threat of any disease‐causing pathogens on animal farms.

Smallholder farmers face challenges in adopting effective biosecurity measures due to limited resources, disparities in knowledge and conflicting awareness of infectious diseases. The challenges include a lack of information about the diseases, zoonotic diseases and vaccination skills. Poor practice of hygiene, access control, waste disposal and pest control also drive disease transmission (Caroline et al. 2017; Jimenez et al. 2023; Munyaneza et al. 2025). The opportunities for change include targeted educational programmes, veterinary services, resource provision, community engagement and digital assistance. Through closing such knowledge gaps and providing appropriate support, smallholder farmers can improve their biosecurity practices, protect their animals and promote overall public and animal health. Through closing such knowledge gaps, they can significantly improve their practices and promote overall public and animal health (Holloway 2019; Otieno et al. 2023; Munyaneza et al. 2025).

In Ethiopia, similar to the case in sub‐Saharan African countries, the few studies that have been conducted have revealed that biosecurity measures are not properly implemented (Otieno et al. 2023; Vougat Ngom et al. 2024). Reliable and exhaustive information is scarce on the biosecurity status of livestock farms (Waktole et al. 2023). A deeper comprehension of the adoption of biosecurity and the obstacles encountered by smallholder farmers in implementing it includes those of a socio‐economic and literacy nature (Chowdhury et al. 2023; Gomez and Mbaga 2023). Despite its importance, biosecurity in Bahir Dar city, Ethiopia, has been poorly conducted, prompting the need for this study to assess the awareness and acceptance of biosecurity practices among small‐ and medium‐sized farmers. Therefore, the study was conducted to assess smallholder farmers’ knowledge and practice towards farm biosecurity measures and associated factors among smallholder farmers of Bahir Dar city, Ethiopia.

## 2 Materials and Methods

### 2.1 Description of Study Area

**FIGURE 1:**
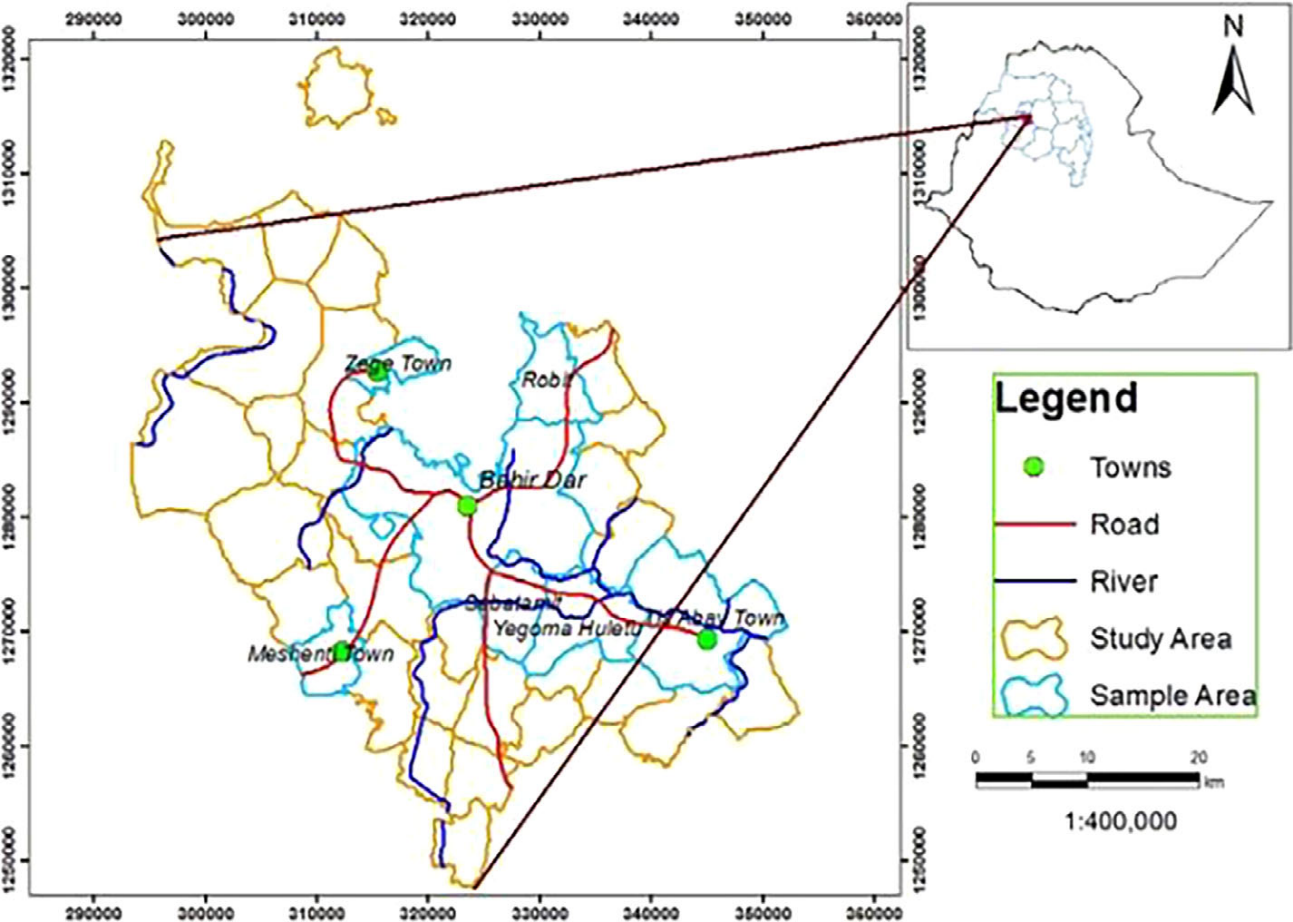
A map indicating the location of the study area, Ethiopia (ARLFDA 2024).

This study was carried out in Bahir Dar city. Bahir Dar is a city located in Northwest Ethiopia at the southern extreme of Lake Tana, where the Blue Nile starts. It is situated at an altitude of about 1820 m above sea level, at the geographic coordinates 11°36′ N and 37°25′ E (Figure 1). The city is 567 and 465 km northwest of Addis Ababa through Debre Markos–Bure and Dejen–Motta roads, respectively. Based on the 2017/18 budget annual statistical bulletin of the Amhara National Regional State Plan Commission, the city administration has a projected population of 341,608 (161,758 males and 179,850 females) (CSA 2017). From this population, 281,886 (82.5%) are urban inhabitants, and the rest, 59,720 (17.5%), are rural inhabitants. The city has a total area of 361.74 km^2^ or 36,174.36 ha. According to the information gathered from the city mayor's office, the city administration is currently divided into six sub‐cities, three satellite towns and 26 urban and 14 rural ‘kebeles’ for administrative purposes (a *kebele* is the smallest administrative unit in Ethiopia) (ARLFDA 2024).

### 2.2 Study Population

All region persons (>18 years of age) residing in Bahir Dar city were a source population, whereas all adult persons (>18 years of age) residing in the randomly selected administrative areas of Bahir Dar city were a study population. One adult person (>18 years of age) from each selected household/family was taken as a study unit. A person (>18 years of age) who resided in a selected household/family and who worked on livestock farms was included in the study. However, a person who resided in selected household/family highly ill or a person unable to give the necessary information at the time of data collection was excluded in the study.

### 2.3 Study Design

A cross‐sectional study was carried out from January 2024 to September 2024 to study the on‐farm biosecurity practices of smallholders, small‐scale farmers who manage areas varying from less than 1 to 10 ha (FAO 2012), located in Bahir Dar city, Ethiopia and identifies variables influencing the overall assessment of biosecurity status.

### 2.4 Sample Size Determination and Sampling Method

#### 2.4.1 Sample Size Determination

Sample size was determined using the formula by Yamane (1967) that is used for small populations. Our pre‐assessment nearly 182 permanently working and registered as tax‐paying smallholder farmers in the study city. Accordingly,

$$n=N/\left(1+Ne^{2}\right)$$

where *n* is the required sample size, *N* is the study population, and *e* is the error of margin; for the study whose confidence level is 95%, *e* = 5%. Thus, the overall sample size was 125 participants, who were selected randomly using a lottery system.

#### 2.4.2 Sampling Methods

The random sampling method was being employed to select the *kebele* of Bahr Dar city and respondents. Before the commencement of the study, preliminary data on livestock farms (occupational community) were sourced from Bahir Dar city. After taking a list of sub‐cities and *kebeles*, livestock producers (dairy, beef, poultry, sheep and goat) were stratified on the basis of their farm specialization. Then from each *kebele*, households having poultry, dairy farms, beef farms and shoats were selected using a simple random sampling technique.

### 2.5 Data Collection Tools and Procedures

#### 2.5.1 Questionnaire Survey

A pretested structured/closed‐ended questionnaire was used for this study. The data were collected by the researchers via interview. The questionnaire was initially developed in English and then translated into Amharic language for appropriateness and ease of communication with the study participants and retranslated to English by different persons to ensure that the meaning was consistent.

Ethical clearance obtained from Bahir Dar University demonstrated the approval of the work. During the interview, the purpose of the study was explained. After a thorough explanation of the objectives and relevance of the study, the procedure, benefits and their rights, informed consent was obtained from the participants and awareness was created. Data were collected after full informed verbal consent was obtained.

#### 2.5.2 Observation Checklist and a Biosecurity Score

Structured observation checklist and biosecurity score were established to identify strengths and weaknesses in current practices of Bahir Dar city. The checklist included categories, such as facility security, personnel practices, equipment maintenance, waste management, animal and animal health management and community engagement, with specific items tailored to local biosecurity threats (Gelaude et al. 2014). A scoring system used for quantifying observations on a scale of 1–5, allowing for the assessment of compliance levels (Mutua et al. 2022).

### 2.6 Operational Definitions

#### 2.6.1 Knowledge Level Related to Farm Biosecurity

A total of 72 questions were asked for each respondent regarding knowledge and practices of farms that minimize disease risks, ensuring healthy livestock, crops and overall farm sustainability. Responses were scored as correct (1) or incorrect (0). The number of questions for which the respondent gave correct responses was counted and scored. This score was then pulled together, and the mean score was computed to determine the overall knowledge of respondents:

$$\begin{array}{c} -\mathrm{Good}\mathrm{knowledge}=\mathrm{respondents}\mathrm{who}\mathrm{score}\geq \mathrm{mean}\mathrm{value}\\ -\mathrm{Poor}\mathrm{knowledge}=\mathrm{respondents}\mathrm{who}\mathrm{score}<\mathrm{mean}\mathrm{value} \end{array}$$

#### 2.6.2 Practice Level Related to Farm Biosecurity

A farm that gained ≥50% was considered having good biosecurity practices, and <50% as poor biosecurity practice (Ahmed et al. 2025).

### 2.7 Data Management and Statistical Analysis

All collected data were entered into a Microsoft Excel spreadsheet, cleaned and coded. Variables that were assumed to have a similar influence on the potential risk of introduction of contagious disease on the farm were combined into a single variable by producing a basic biosecurity score (Van Steenwinkel et al. 2011). The dependent variables were a dichotomous variable of good/poor knowledge status and good/poor practice status. Independent variables are residence district, sex, age, residence *kebele*, educational status, occupation, livestock farming experience (years), previous experience on farm biosecurity, previous training in biosecurity, sources of premises, farm capacity and farm type. Presence of record keeping, presence of isolation room for diseased animals, vaccinating chickens for diseases known in the past, vaccinating of animals according to the manufacturer's instructions, using antibiotics only for sick animals, using antibiotics according to the recommended dosage and not using expired vaccines/drugs.

Statistical analyses were performed using STATA, version 13 statistical software. Multivariable logistic regression analysis was used to estimate associations between the demography of smallholder farm owners and farm characteristics with biosecurity status. A variable is said to have a significant effect when *p* < 0.05.

### 2.8 Ethical Consideration

The study followed the Declaration of Helsinki ethical guidelines for research on human subjects. Research ethics is one of the foundations of modern‐day scientific research in any area of study (Barrow et al. 2025). Ethical clearance letters were obtained from Bahir Dar University, College of Agriculture and Environmental Sciences (Ref. No. 2/2210/1‐1‐5) before conducting the research. In addition, permission was also sought from the Amhara Regional Livestock Agency and the city Livestock Office to conduct the study. The interview began with a clarification of the study purpose. After a clear explanation of the objective and significance of the study, procedure, benefit and their rights, informed consent was taken from the participants. Data were collected after informed verbal consent. The final report was presented and submitted in hard copy to the department of veterinary science, with a summary of the study's findings and conclusions.

## 3 Results

### 3.1 Socio‐Demographic Characteristics of Farm Owners

A total of 125 household farmers with different livestock population sizes were visited during this survey. The majority of farm owners were male, 103 (82.4%). Most farm owners, 75 (60%), have previous experience with farm biosecurity. A little more than half of the farm owners, 68 (54.4%), have received training in biosecurity (Table 1).

**TABLE 1:** Socio‐demographic characteristics of farm animal owners involved in biosecurity evaluation.

Variables (farm owners’ demography)	Parameters (category)	Number	Percentage
Gender	Female	22	17.6
	Male	103	82.4
Age group (years)	20–30	23	18.4
	31–45	73	58.4
	>45	29	23.2
Education level	Illiterate (no formal education)	51	40.8
	Primary and secondary school	53	42.4
	Higher education	21	16.8
Primary occupation	Trader	69	55.2
	Profession^a^	56	44.8
Livestock farming experience (years)	<4	18	14.4
	>4	95	76
	Prefer not to answer	12	9.6
Previous experience on farm biosecurity	Yes	75	60
	No	50	40
Previous training in biosecurity	Yes	68	54.4
	No	57	45.6

^a^ **Professional groups,** such as veterinarians, higher education and agricultural extension workers, and those who have their own farm.

### 3.2 Characteristics of Farms

The ownership structure of the farm premises was categorized by sources of premises, farm capacity and farm type as indicated by Table 2. As to the type of farming, poultry farming dominated with 62 (49.6%).

**TABLE 2:** Farms with various farm characteristics (*n = *125).

Characteristics	Category	Number	Percentage
Sources of premises	Owned	72	57.6
	Rented	53	42.4
Farm capacity	Small‐scale	88	70.4
	Medium‐scale	37	29.6
Farm type	Poultry	62	49.6
	Diary	28	22.4
	Beef	18	14.4
	Shoat	17	13.6

### 3.3 Variables of Farm Biosecurity

As presented in Table 3, the data tell variation in farm features and biosecurity practices with the main routes for improvement in employee training and structural biosecurity measures. Among the farms, 57 (45.6%), 77 (61.6%) and 81 (64.8%) were greater than 200 m far from the main road, have the biosecurity plan and provide biosecurity planning training for the employee, respectively.

**TABLE 3:** Indicators of farm biosecurity (*N* = 125).

Variable (biosecurity indicators)	Category	Number	Percentage
Distance of the farm from the main road (m)	70–100	28	22.4
	100–200	40	32
	>200	57	45.6
Distance from the nearest farm (m)	<100	41	32.8
	≥100	84	67.2
Distance from the residential place (m)	0–60	49	39.2
	>60–100	56	44.8
	>100	20	16
No standing water near the farm	Yes	101	80.8
	No	24	19.2
Premise with modified open‐side and curtains	Yes	62	49.6
	No	63	50.4
Housing position	East–west	78	62.4
	North–south	47	37.6
Presence of the biosecurity plan	Yes	77	61.6
	No	48	38.4
Biosecurity training to employee	Yes	44	35.2
	No	81	64.8
Separate delivery of purchased animals	Yes	62	49.6
	No	63	50.4

### 3.4 Farm Biosecurity Knowledge of Respondents

For the assessment of the structural biosecurity, 21 biosecurity measurements were considered (Table 4). A total of 108 (86.4%) respondents were exchanging equipment with other farms. About 93 (74.4%) of farms restrict visitor entry, which decreases the possibility that any pathogens will be introduced from the outside. Being up‐to‐date with regard to outbreaks of farm diseases. A total of 49.6% of farms showed an important risk history of disease outbreaks, enhancing the need for strict biosecurity practices.

**TABLE 4:** Knowledge of respondents about farm biosecurity measures in Bahir Dar city, Ethiopia (*N* = 125).

Variable	Response	Number	Percentage
Equipment exchange with another farm	Yes	108	86.4
	No	17	13.6
Presence of functional footbath	Yes	78	62.4
	No	47	37.6
Farm vehicle parked off the farm	Yes	105	84
	No	20	16
Presence of fence and gate	Yes	102	81.6
	No	23	19.4
Prohibition of vehicle entry	Yes	105	84
	No	20	16
Presence of tyre bath/spray at the gate	Yes	21	16.8
	No	104	83.2
Prohibition of entry of visitors	Yes	93	74.4
	No	32	25.6
Stay informed regarding outbreak of farm disease in the area	Yes	65	52
	No	60	48
Surface water not used for cleaning	Yes	102	81.6
	No	23	19.4
Store feed in an area protected from contamination	Yes	99	79.2
	No	26	21.8
Have a relationship with a herd veterinarian?	Yes	48	38.4
	No	77	61.6
Have an animal health management plan?	Yes	71	56.8
	No	54	43.2
Experience of disease outbreaks on the farm	Yes	62	49.6
	No	63	50.4
Purchase feed from suppliers	Yes	72	57.6
	No	53	42.4
Keep feed records on the source	Yes	68	54.4
	No	57	45.6
Remove and renew bedding on a regular schedule	Yes	104	83.2
	No	21	16.8
Have walls, ceilings and facility parts that are easy to clean and disinfect	Yes	61	48.8
	No	64	51.2
Can name any biosecurity measure that can help prevent the spread of diseases among livestock?	Yes	47	37.6
	No	78	62.4
Surface water not used for drinking of animal	Yes	93	74.4
	No	32	25.6
	Yes	59	47.2
Are you familiar with the concept of farm biosecurity? Reporting notifiable diseases in livestock?	No	66	52.8
	Yes	55	44
	No	70	56
**Overall farm biosecurity knowledge of respondents**	Status	Frequency	Percentage
	Good	50	40
	Poor	75	60

This study revealed the overall biosecurity status for each farm. Thus, 50 (40%) farms have got a score of >50%; therefore, their biosecurity knowledge was presented as good. The remaining 75 (60%) farms scored <50%; hence, their knowledge was graded as poor.

### 3.5 Farm Biosecurity Practices of Respondents

As presented in Table 5, farm workers of 50 (40%) farms did not wear special clothes, and 65 (52%) of farms practised regular cleaning and disinfection. Among the respondents, 89 (71.2%) use special footwear, but 91 (72.8%) were not using special face‐masker. From the participants, 101 (80.8%) were not testing the drinking water for bacterial contamination, and 85 (68%) were not practising a record keeping.

**TABLE 5:** Farm biosecurity practice measures in Bahir Dar city, Ethiopia (*N* = 125).

Variable	Response	Number	Percentage
Use of special cloth	Yes	75	60
	No	50	40
Use of special footwear	Yes	89	71.2
	No	36	28.8
Use of special masker	Yes	34	27.2
	No	91	72.8
Use of special hat	Yes	38	30.4
	No	87	69.6
Shower in and out	Yes	36	28.8
	No	89	71.2
Regular laundering to cape and coveralls	Yes	44	35.2
	No	81	64.8
Order of care is from youngest to oldest	Yes	63	50.4
	No	62	49.6
Share their knowledge of biosecurity measures with other farmers	Yes	73	58.4
	No	52	41.6
Main challenges faced in implementing biosecurity measures on your farm?	No relationship with a herd veterinarian	26	20.8
	Lack of knowledge about farm biosecurity	42	33.6
	Financial problems	57	45.6
Perform post mortems for unexplained deaths to monitor disease?	Yes	40	32
	No	85	68
Test drinking water for bacterial contamination?	Yes	24	19.2
	No	101	80.8
Group animals by susceptibility to disease	Yes	58	46.4
	No	67	53.6
Presence of record keeping	Yes	40	32
	No	85	68
Staff has no contact with other farms	Yes	50	40
	No	75	60
Regular cleaning and disinfection	Yes	65	52
	No	60	48
How often do you clean and disinfect your livestock housing areas and equipment?	Once a week	81	64.8
	Once a month	44	35.2
Do you practice proper manure management to reduce disease risks?	Yes	63	50.4
	No	62	49.6
Monitor the health of livestock for signs of illness?	Take animal to nearby animal clinic	79	63.2
	Try to medicate animal by local knowledge	46	36.8
Encountered any disease outbreaks and informed to a veterinary?	Yes	73	58.4
	No	52	41.6
Do you feel adequately equipped with knowledge and resources to implement biosecurity measures on your farm?	Yes	42	33.6
	No	83	66.4
How important do you think biosecurity measures are for the health and productivity of your livestock?	Highly important	81	64.8
	Less important	44	35.2
Do you believe that implementing biosecurity measures can help prevent disease outbreaks on your farm?	Yes	87	69.6
	No	38	30.4
Presence of isolation room for diseased animals	Yes	47	37.6
	No	78	62.4
Separation of material for young and old animals	Yes	80	64
	No	45	36
Use equipment for a single purpose only	Yes	77	61.6
	No	48	38.4
Dispose of dead stock by burying, composting or pickup by a dead‐stock disposal service	Yes	65	52
	No	60	48
Practice sanitation to minimize contamination of livestock waters by manure and urine	Yes	93	74.4
	No	32	25.6
Vaccinating animals for diseases known in the past	Yes	98	78.4
	No	27	21.6
Vaccinating animal according to the manufacturer's instruction	Yes	84	67.2
	No	41	32.8
Using antibiotics only sick animals	Yes	79	63.2
	No	46	36.8
Using antibiotics according to the recommended dosage	Yes	78	62.4
	No	47	37.6
Not using expired vaccines/drug	Yes	89	71.2
	No	36	28.8
**Practices of farm biosecurity**	Status	Frequency	Percentage
	Good **Practices**	62	49.6
	Poor **Practices**	63	50.4

This study revealed the overall biosecurity status for each farm. Thus, 62 (49.6%) farms have got a score of >50%; therefore, their biosecurity practices were presented as good. The remaining 63 (50.4%) farms scored <50%; hence, their practices were graded as poor.

### 3.6 Factors Affecting Good Farm Biosecurity Knowledge and Practices

#### 3.6.1 Factors Affecting Good Farm Biosecurity Knowledge

Our multivariable analysis showed that gender, educational level and main occupation status were found significant risk factors associated with good level of farm biosecurity knowledge. Among the respondents’ male farm owners had good knowledge about farm biosecurity (adjusted odds ratio [AOR] = 1.43, 95% confidence interval [CI] 1.12–2.56, *p* = 0.005) than female farm owners. The odds of having good knowledge were 2.05 times higher of college‐ and university‐level educated farmers (AOR = 2.05; 95% CI 1.56–1.99, *p* = 0.000) than illiterate ones. Likewise, those respondents who are traders, had poor knowledge about farm biosecurity (AOR = 3.79; 95% CI 1.05–1.54 *p* = 0.000) compared with those professionals’ farm owner respondents. Traders are 3.79 times less likely to experience the good knowledge about farm biosecurity compared with professionals (Table 6).

**TABLE 6:** Risk factors affecting the good knowledge about farm biosecurity based on multivariable logistic regression in and around Bahir Dar city (*N* = 125).

I. Knowledge		Knowledge level score				
Variable	Category	Good	Poor	COR (95% CI)	AOR (95% CI)	*p* value
**Gender**	Female	6 (12%)	16 (21.3%)	Ref.	Ref.	Ref.
	Male	44 (88%)	59 (78.6%)	2.71 (0.49–0.98)	1.43 (1.12–2.56)	0.005
**Age group (years)**	20–30	20 (27.7%)	3 (5.6%)	Ref.	Ref.	Ref.
	31–45	30 (41.6%)	44 (83%)	0.99 (1.92–2.21)	1.47 (2–3.43)	0.557
	>45	22 (30.5%)	6 (11.3%)	1.32 (0.45–2.32)	3.16 (0.43–2.12)	0.955
**Education level**	Illiterate (no formal education)	10 (16.3%)	34 (53.1%)	Ref.	Ref.	Ref.
	Primary and secondary school	21 (34.4%)	23 (35.9%)	0.76 (0.21–1.41)	2.55 (0.02–1.65)	0.786
	Higher education	30 (49.1%)	7 (10.9%)	3.01 (1.31–2.10)	2.05 (1.56–1.99)	0.000
**Primary occupation**	Trader	44 (59.4%)	25 (49%)	Ref.	Ref.	Ref.
	Profession^a^	30 (40.5%)	26 (50.9%)	4.78 (1.03–1.54)	3.79 (0.05–1.45)	0.000
**Livestock farming experience (years)**	<4	10 (15.1%)	8 (13.55)	Ref.	Ref.	Ref.
	>4	51 77.2%)	44 (74.5%)	3.32 (0.27–1.87)	4.60 (0.28–1.01)	0.797
	Prefer not to answer	5 (7.55)	7 (11.8%)	0.45 (0.55–1.79)	1.54 (0.11–1.98)	0.085
**Previous experience on farm biosecurity**	Yes	48 (57.2%)	27 (65.8%)	8.12 (0.09–1.20)	9.01 (0.29–2.04)	0.146
	No	36 (42.8%)	14 (34.1%)	Ref.	Ref.	Ref.
**Previous training in biosecurity**	Yes	51 (60.7%)	17 (41.4%)	3.21 (0.76–2.91)	4.23 (0.04–1.54)	0.634
	No	33 (39.2%)	24 (58.9%)	Ref.	Ref.	Ref.

Abbreviations: AOR, adjusted odd ratio; CI, confidence interval; COR, crude odds ratio.

^a^ **Professional groups,** such as veterinarians, higher education and agricultural extension workers, have farms.

Other variables, such as age group (years), livestock farming experience (years), previous experience on farm biosecurity, previous training in biosecurity, sources of premises and farm capacity and farm type, were not statically associated.

#### 3.6.2 Factors Affecting Good Farm Biosecurity Practices

Our multivariable analysis indicated that gender, education level and primary occupation were also factors for good farm biosecurity practice. Male farm owners are approximately 1.34 times more likely to apply good farm biosecurity practice than female farm owners (AOR = 1.34; 95% CI 1.06–1.36, *p* = 0.001). The odds of having good farm biosecurity practices were 2.05 times higher for education‐level farmers (AOR = 2.05; 95% CI 1.31–2.10, *p* = 0.000) than for illiterate ones. Another predictor detected in our analysis was primary occupation; professionals have (AOR = 1.071; 95% CI 1.01–1.87, *p* value = 0.004). Traders have about 1.071 times less likely to adhere to good farm biosecurity practice than professionals. The remaining other variables tested were not associated with good farm biosecurity practice (Table 7).

**TABLE 7:** Risk factors affecting the good farm biosecurity practice based on multivariable logistic regression in and around Bahir Dar city (*N* = 125).

I. Practice		Practice level score				
Variable	Category	Good	Poor	COR (95% CI)	AOR (95% CI)	*p* value
**Gender**	Female	6 (9.6%)	16 (25.3%)	Ref.	Ref.	Ref.
	Male	56 (90.45%)	47 (74.6%)	1.92 (2.49–3.48)	1.34 (1.06–1.36)	0.001
**Age group (years)**	20–30	9 (16.1%)	7 (14.8%)	Ref.	Ref.	Ref.
	31–45	29 (51.7%)	38 (80.8%)	0.08 (1.02–2.51)	2.47 (0.76–3.93)	0.557
	>45	18 (32.1%)	2 (4.2%)	2.32 (0.85–2.02)	4.28 (0.43–4.92)	0.955
**Education level**	Illiterate (no formal education)	19 (26.7%)	32 (59.2%)	Ref.	Ref.	Ref.
	Primary and secondary school	35 (49.2%)	18 (33.3%)	0.06 (0.61–1.01)	1.55 (0.02–4.65)	0.786
	Higher education	17 (23.9%)	4 (7.4%)	3.01 (1.31–2.10)	2.05 (1.31–2.10)	0.000
**Primary occupation**	Trader	36 (37.5%)	20 (68.9%)	Ref.	Ref.	Ref.
	Profession	60 (62.5%)	9 (31.5%)	1.35 (2.03–4.54)	1.071 (1.01–1.87)	0.004
**Livestock farming experience (years)**	<4	10 (11.3%)	8 (21.6%)	Ref.	Ref.	Ref.
	>4	71 (80.6%)	24 (64.8%)	4.21 (0.17–0.47)	4.60 (0.28–2.31)	0.884
	Prefer not to answer	7 (7.9%)	5 (13.5%)	1.45 (0.95–2.99)	2.54 (0.03–1.78)	0.985
**Previous experience on farm biosecurity**	Yes	52 (74.2%)	23 (41.8%)	9.12 (0.69–1.91)	6.31 (2.06–3.74)	0.046
	No	18 (25.7%)	32 (58.2%)	Ref.	Ref.	Ref.
**Previous training in biosecurity**	Yes	56 (73.6%)	12 (24.4%)	2.41 (0.56–2.51)	4.03 (0.14–6.04)	0.534
	No	20 (26.3%)	37 (7.5%)	Ref.	Ref.	Ref.
**Sources of premises**	Owned	49 (58.3%)	23 (56%)	Ref.	Ref.	Ref.
	Rented	35 (41.6%)	18 (44%)	4.05 (0.67–2.54)	7.90 (0.77–3.65)	0.758
**Farm capacity**	Small‐scale	64 (80%)	24 (53.33%)	Ref.	Ref.	Ref.
	Medium‐scale	16 (20%)	21 (46.67%)	0.99 (0.45–2.67)	8.01 (0.33–6.31)	0.527
**Farm type**	Poultry	43 (52.2%)	19 (46.4%)	Ref.	Ref.	Ref.
	Diary	20 (23.8%)	8 (19.5%)	6.36 (0.27–1)	10 (0.40–4.01)	0.060
	Beef	12 (14.3%)	6 (14.6%)	0.89 (0.67–1.43)	2.02 (0.22–3.10)	0.876
	Shoat	9 (10.7%)	8 (19.5%)	3.23 (0.11–1.21)	4.43 (0.21–1.34)	0.213

Abbreviations: AOR, adjusted odd ratio; CI, confidence interval; COR, crude odds ratio.

## 4 Discussion

The demographic profile of the farm owners provides fascinating information about biosecurity behaviour within and around Bahir Dar city. Respondents in our study area were predominantly male (82.4%), as is the case with broader agricultural trends in Ethiopia, where men are more active in agriculture (Neway and Zegeye 2022). With regard to the level of education, it reflected an interesting trend where 42.4% of respondents had a primary or secondary level of education. This limited level of educational attainment can exclude acknowledgement and efficient application of biosecurity practices, as previous research in Ethiopia and elsewhere emphasizes the reality that higher education contributes to considerable promotion of improved agricultural and animal health practices (Nyokabi et al. 2023). Training and experience are also valuable; although 60% of the participants had prior biosecurity experience, nearly half (45.6%) had no formal training, supporting the need for highly structured educational interventions to improve knowledge and practice compliance with biosecurity best practices.

Farm features also pose important biosecurity risks in our study area. Most farms (70.4%) were small and typically situated near main roads (<70 m) or along neighbouring farms (<100 m). These locations enable the transmission of airborne diseases between farms and from incoming animals, with studies showing that a buffer area of more than >100 m is necessary to prevent this occurrence (Gelaude et al. 2014). Similarly, 60% of smallholder farms were not more than 60 m distant from dwelling estates and threatened pollution, zoonotic spillover and financial losses (KA and Benson 2014). Moreover, the disposal of dead animals was a shortcoming, with 48% of farmers not disposing of their dead animals in healthy conditions, and 64.8% of them were not regularly washing their protective equipment. All these activities create enormous reservoirs for infectious pathogen transmissions and are used as incubation centres (Van Limbergen et al. 2018; Wijesinghe et al. 2017).

Regarding structural biosecurity, 62.4% of farms in this study had a footbath at the gate, and this was higher than the finding in Bishoftu town, Ethiopia where 40% (Ismael et al. 2021) and Bali, Indonesia, where 38% of the farms had footbaths at the farm gate (Ambarawati et al. 2011). The feet of humans, animals and vehicles have significant risks, as they are well‐entrenched mechanical carriers of pathogens (McQuiston et al. 2005). The management of visitors was relatively strong in our study, with 74.4% of farms regulating who could visit, a practice supported by international guidelines to prevent human‐mediated transmission of pathogens (Hagenaars et al. 2018; England 2002). Yet, reliance on foreign feed sources is also a vulnerability, as feed and transport vehicles for many infectious diseases, such as *Salmonella* spp.*, Escherichia coli, Clostridium* spp.*, Aspergillus* spp. and mycotoxins, in a variety of locations throughout the chain of production and storage (Lister 2008).

In our study area, smallholder farmers have the challenge of practising effective biosecurity measures due to scarcity of resources, knowledge gaps and reliance on usually traditional livestock farming practices. Even though there are some farmers who value the importance of biosecurity, most of them have limitations with the application, particularly for activities such as isolation of sick animals, quarantine and hygienic practices. More access to information and support tailored to them is required to improve biosecurity uptake and reduce disease risk within smallholder farming systems (Holloway 2019; Chenais et al. 2023; Otieno et al. 2023; Munyaneza et al. 2025). This study's findings revealed that nearly half of major biosecurity activities were not done of the surveyed farms. A total of 62 (49.6%) farms scored above 50%, indicating adherence to good biosecurity practices aimed at minimizing disease risks and safeguarding livestock health. In contrast, 63 (50.4%) farms scored below 50%, denoting poor biosecurity measures, which could lead to vulnerabilities in disease exposure. This finding was in‐line with results of research conducted in Belgium (Gelaude et al. 2014) and other regions, where high acquiescence variation suggests the need for specific strategies and policy to enhance biosecurity in our study area; because of that, disease prevention is more important than disease treatment interventions.

Logistic regression analysis also explained the effects of socio‐demographic characteristics on the effective application of biosecurity measures on their farm. Education, occupation and gender significantly affected biosecurity practice and knowledge. Male (AOR = 1.34; 95% CI 1.06–1.36, *p* = 0.001) farmers were more likely to adopt good practices than females. The women who are typically disadvantaged in access to education, training and agricultural extension thus had less knowledge (Angasu et al. 2023; Davis et al. 2010). Education level was one of identified predictors of our study area, with those who had higher education (learned farmers) (AOR = 2.05; 95% CI 1.31–2.10, *p* = 0.000) being 2.05 times more likely to engage in good practices than illiterate (no formal education) farmers; this reflects their reliance on customary practice and reduced their awareness of preventive practices (Tsegaye et al. 2023; Hailemariam et al. 2017). Occupation type was also relevant: Traders and informal workers had worse biosecurity uptake than professional groups, such as veterinarians, higher education and agricultural extension workers (AOR = 1.071; 95% CI 1.01–1.87, *p* value = 0.004), who typically undergo specialized training (Moje et al. 2023). This our study finding was in‐line with earlier Ethiopian studies blaming demographic variation for biosecurity uptake variability (Gebre et al. 2021; Nyokabi et al. 2023; Kabeta et al. 2024; Munyaneza et al. 2025).

Cumulatively, our study evidence suggests that demographic pressures, risky farm location and lack of proper application of structural and operational controls put smallholder farms in Bahir Dar city at risk of disease introduction and transmission. In addition, in Ethiopia, particularly in our study area region, absence of a policy and strategy for farm biosecurity measures can lead to significant risks of disease occurrences. Disease can cause devastating consequences for farmers, livestock welfare and the wider food supply chain of the area (Kappes et al. 2023). Related with occurrences of disease, farmers’ misuse of drugs in animal production contributes to increasing antimicrobial resistance. If no biosecurity measures are taken, the effectiveness of usual antimicrobials designed to treat acute or chronic infections may be under question. Moreover, improperly keeping biosecurity may lead to the spread of zoonotic infection from animals to humans. On the other hand, effective adoption of biosecurity practices may cause improved animal health, enhanced quality of animal products, reduced risks and maintaining a healthy environment, disease prevention, protecting animal welfare and long‐term cost savings for smallholder farmers (WOAH 2017; Kappes et al. 2023; Moiane 2025). These show the need for addresses by joint interventions, including more equal access for female and low‐educated farmers to biosecurity training, developing policies and strategies for farm biosecurity and more efficient extension services that are tailored for traders and small‐scale players. These measures would enhance farm‐level resilience, reduce zoonotic spillover risks and safeguard animal and public health (Gill et al. 2025; Omuse et al. 2025).

### 4.1 Limitation of the Study

- Sample size of the study was narrow because it was difficult to go and study a large sample size due to the current condition.

## 5 Conclusions

Overall farm biosecurity knowledge of respondents and biosecurity practice levels of the study farms were below half expected biosecurity activity scores. The findings from this study suggest that the practice of biosecurity implementation strategies in household farms in Bahir Dar needs to be improved through interventions. The majority of the biosecurity risks for stallholder farms originate from inappropriate site selection and feed sources as well as lack of training to farm employees. Our multivariable analysis indicated that gender, occupation and education were factors for good farm biosecurity knowledge and practices among smallholder farm respondents of the study area. The remains of other variables tested were not associated with good farm biosecurity practice. Thus, awareness should be created for farm owners and workers towards modern farm biosecurity practices for improvement of production and productivity and sustainable growth of the animals of smallholder farms. Interventions such as training, workshops and field day seminars by appropriate agents on the benefit of adhering strictly to biosecurity measures on their farms. Effective and efficient disease control and prevention strategies should be conducted in smallholder farms by implementing innovative biosecurity activities. Those farm owners should enhance the biosecurity management system by establishing strong linkages with veterinarians. Educated professionals should be encouraged to involve themselves in smallholder farm production because they have good farm biosecurity knowledge and practices. Further intensive research should be conducted to develop supportive policies and for the development of innovative solutions to implement appropriate farm biosecurity measures.

## Author Contributions

**Birhan Agmas Mitiku**: conceptualization, methodology, resources, supervision, visualization, writing – original draft, writing – review and editing. **Ayele Adugna Gedifew**: conceptualization, formal analysis, methodology, writing – original draft. **Temsegen Sendekie Ayalew**: methodology, supervision, validation, visualization, writing – review and editing.

## Conflicts of Interest

The authors declare no conflicts of interest.

## Peer Review

The peer review history for this article is available at https://publons.com/publon/10.1002/vms3.70651.

## Data Availability

All information is available upon reasonable request from the corresponding author.
